# Mucosa-associated lymphoid tissue (MALT) lymphoma developing in ectopic mediastinal thyroid tissue: a case report

**DOI:** 10.1186/s40792-020-00857-2

**Published:** 2020-05-11

**Authors:** Naotaka Uchida, Manabu Yoshida

**Affiliations:** 1 Department of Breast, Endocrine and Thoracic Surgery, Matsue City Hospital, 32-1, Noshira-cho, Matsue, Shimane 690-8509 Japan; 2 Department of pathology, Matsue City Hospital, 32-1, Noshira-cho, Matsue, Shimane 690-8509 Japan

**Keywords:** Ectopic mediastinal thyroid, Mucosa-associated lymphoid tissue (MALT) lymphoma, Chronic thyroiditis, Mediastinal mass, Serum levels of interleukin-2 receptor, Immunoglobulin heavy chain class switch recombination

## Abstract

**Background:**

Normally located in the neck, ectopic mediastinal thyroid tissue consists of very rare ectopic thyroid tissue that does not connect to the thyroid gland. A patient with mucosa-associated lymphoid tissue (MALT) lymphoma that has developed in mediastinal thyroid tissue, to our best knowledge, has not been previously reported.

**Case presentation:**

A 67-year-old woman presented with a superior mediastinal mass that was revealed by chest computed tomography (CT), an optional examination she hoped, during a medical checkup. Contrast-enhanced CT scan performed in our hospital for close examination confirmed the presence of a 2 × 1.3 cm poorly enhanced mass anterior to the trachea during the arterial phase. Magnetic resonance imaging depicted low signal intensity on T1-weighted images and high signal intensity on T2-weighted images. I-131 meta-iodobenzylguanidine did not accumulate in the mass. Serum levels of interleukin-2 receptor, catecholamine, and anti-acetylcholine receptor antibody were within the normal range. The mass was resected through a transverse neck incision for the diagnosis and treatment. The histopathological diagnosis of the specimen was ectopic mediastinal thyroid tissue associated with MALT lymphoma and chronic thyroiditis. Immunoglobulin heavy chain class switch recombination was identified. Fine-needle aspiration biopsy of the cervical thyroid showed chronic thyroiditis but not lymphoma. The patient’s postoperative thyroid function was normal. To date, the patient’s recovery has been uneventful, and she is being monitored without further treatment.

**Conclusion:**

We treated the patient with MALT lymphoma that developed in ectopic mediastinal thyroid tissue. This novel case illustrates a new differential diagnosis associated with ectopic mediastinal thyroid tissue.

## Background

Ectopic thyroid tissue is located anywhere other than its usual anatomical position, which is in the neck anterior to the trachea, from the second to the fourth tracheal cartilage. Mediastinal thyroid tissue is very rare, accounting for less than 1% of all cases of ectopic thyroid tissue [1]. The classification of mediastinal thyroid proposed by Rives is generally used as follows: ectopic thyroid tissue is not connected to the cervical thyroid gland, whereas a substernal thyroid is connected to the cervical thyroid gland [2]. Mediastinal thyroids account for 3% of all mediastinal masses, with ectopic mediastinal thyroids accounting for 2% of mediastinal thyroids [3, 4]. Malignant transformation in ectopic mediastinal thyroid tissue is extremely rare [5, 6]. Furthermore, such a malignancy is virtually always diagnosed by a histopathological examination after surgical excision of the lesion [6].

Primary thyroid lymphoma is a rare disease, accounting for about 1 to 2% of all extranodal malignant lymphomas [7]. The most common types of primary thyroid lymphoma diagnosed at Ito Hospital, a high-volume center for thyroid disease in Japan, have been diffuse large B cell lymphoma (DLBCL), occurring in 51% of cases, and mucosa-associated lymphoid tissue (MALT) lymphoma, occurring in 47% of cases [8]. To the best of our knowledge, MALT lymphoma associated with ectopic mediastinal thyroid tissue has not been previously reported.

## Case presentation

**Fig. 1:**
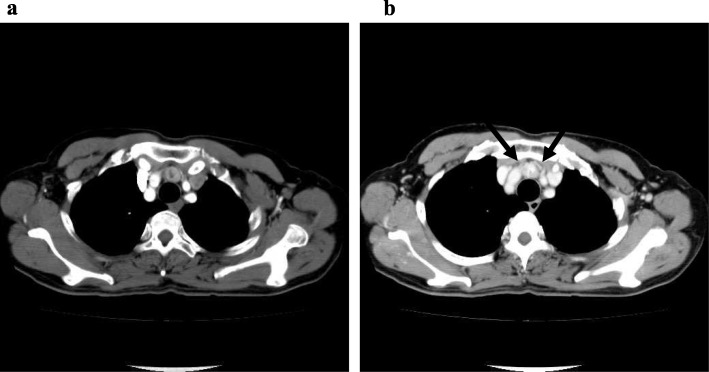
Contrast-enhanced CT images of ectopic mediastinal thyroid tissue. **a** Poor enhancement with a low density of the central region in the early phase. **b** Strong enhancement, especially of the central region, in the delayed phase. Arrows show tumor-feeding arteries arising from the vessels in the thorax

**Fig. 2:**
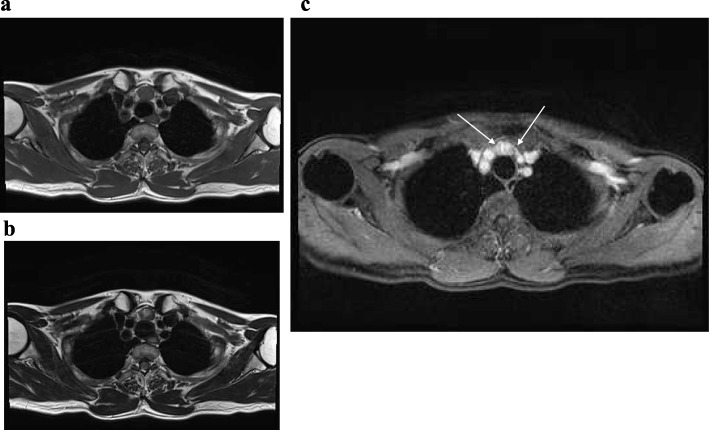
MRI images of ectopic mediastinal thyroid tissue. **a** Low signal intensity on T1-weighted images. **b** High signal intensity on T2-weighted images. **c** Enhanced MRI showing stronger enhancement. Arrows show tumor-feeding arteries arising from the vessels in the thorax

A 67-year-old woman presented with a superior mediastinal mass that was revealed by chest computed tomography (CT), an optional examination she hoped, during a medical checkup. She did not have dyspnea, dysphagia, blepharoptosis, diplopia, general fatigue, fever, night sweats, or weight loss. Contrast-enhanced CT confirmed the presence of a 2 × 1.3 cm mass anterior to the trachea in the superior mediastinum, which was characterized by peripheral poor enhancement and central low density during the arterial phase (Fig. 1a). The central region of the mediastinal mass was enhanced during the secondary phase (Fig. 1b). Tumor-feeding arteries were observed arising from the vessels in the thorax (Fig. 1b). The mediastinal mass was circumscribed and was not connected with the thyroid gland in the neck. Magnetic resonance imaging (MRI) of the mediastinal mass depicted low signal intensity on T1-weighted images (Fig. 2a) and high signal intensity of the central region on T2-weighted images (Fig. 2b). Diffusion-weighted imaging of the mass showed a signal intensity that was similar to that of the thyroid gland. The apparent diffusion coefficient was not decreased. Enhanced MRI of the mass showed stronger enhancement than that of the thyroid gland, and tumor-feeding arteries arising from the vessels in the thorax were observed (Fig. 2c). The radiologist suggested that the mass was a paraganglioma. I-131 meta-iodobenzylguanidine did not accumulate in the mediastinal mass. Serum levels of lactate dehydrogenase, interleukin-2 receptor (sIL-2R), catecholamine, and anti-acetylcholine receptor antibody were within the normal range. Preoperative thyroid function was not assessed.

**Fig 3:**
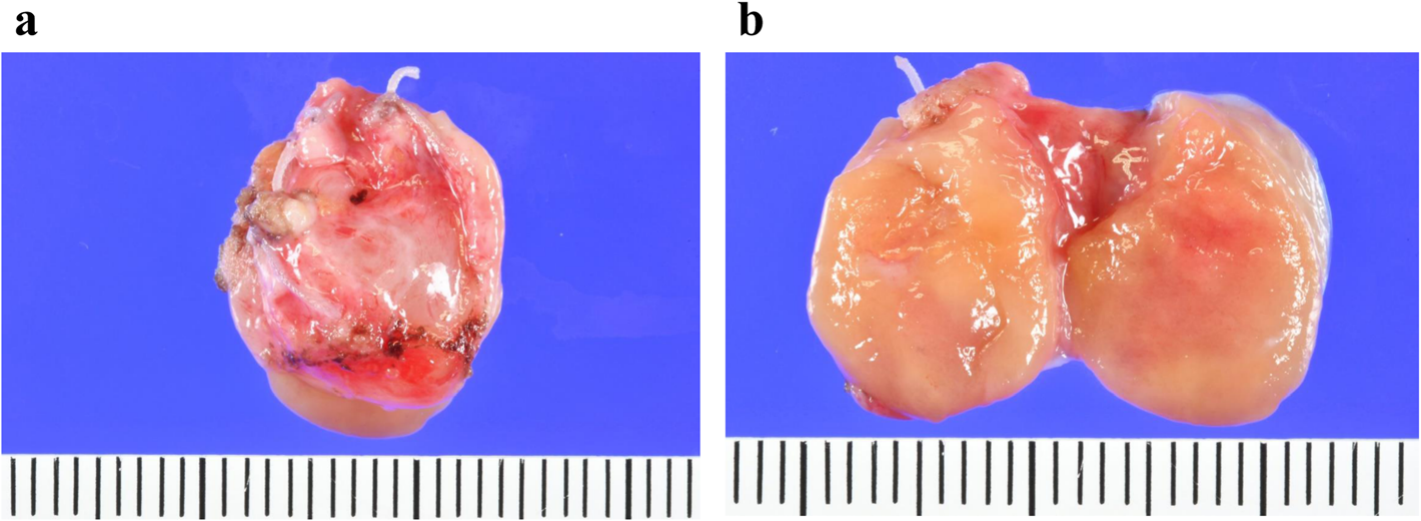
Photos of the resected specimen. **a** The oval, soft tumor, 15 × 12 × 9 mm in size. **b** The split face of the tumor

**Fig. 4:**
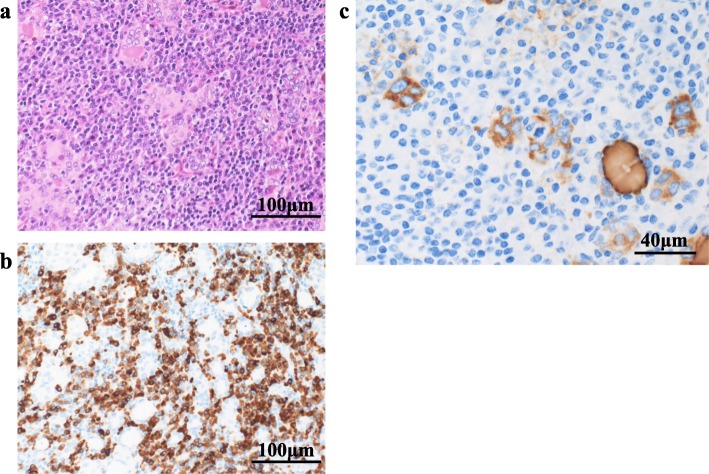
Histopathological findings of ectopic mediastinal thyroid tissue. A horizontal bar indicates the actual length. **a** Abundant thyroid follicles and lymphocytes with hematoxylin-eosin staining. **b** Positive lymphocytes on CD79a staining. **c** Lymphoepithelial lesion to epithelial cells stained positive for thyroglobulin

For diagnosis and treatment, the patient underwent resection of the mass through a transverse neck incision. The well-circumscribed mass was located anterior to the trachea. The cervical thyroid appeared to be normal and was not connected to the mediastinal mass, which had an independent blood supply from the vessels in the thorax. A frozen section of the mass showed ectopic thyroid tissue and chronic thyroiditis. The resected specimen was 15 × 12 × 9 mm and opaline in color (Fig. 3a, b). Histopathological examination of the specimen found abundant thyroid follicles and lymphocytes, suggesting a lymphoproliferative disorder (Fig. 4a). The lymphocytes stained positive for cluster of differentiation (CD)79a (B cell marker) (Fig. 4b). The lymphoepithelial lesion that showed lymphoma cell infiltration to epithelial cells stained positive for thyroglobulin, a characteristic of MALT lymphoma arising from ectopic thyroid tissue (Fig. 4c). Lymphocytes in the mass were prominent peripherally. Polymerase chain reaction identified recombination of the DH1-6/JH region of the immunoglobulin heavy chain, indicating immunoglobulin heavy chain class switch recombination (Table 1). Therefore, the diagnosis was ectopic thyroid tissue associated with MALT lymphoma due to chronic thyroiditis. 18F-fluoro-deoxyglucose positron emission tomography (FDG-PET) showed a marked accumulation in the cervical thyroid, but no accumulation elsewhere. A fine-needle aspiration biopsy of the cervical thyroid gland that was performed postoperatively showed chronic thyroiditis but no evidence of lymphoma. Postoperative thyroid function and the sIL-2R level were within the normal range. The serum levels of anti-thyroglobulin antibody and anti-peroxidase antibody assessed after surgery were elevated at 33 IU/mL (normal range, 0–27 IU/mL) and at 210 IU/mL (normal range, 0.0–15.9 IU/mL), respectively. The patient has been monitored with no further treatment. She has been well with no recurrence thus far.

**Table 1:**
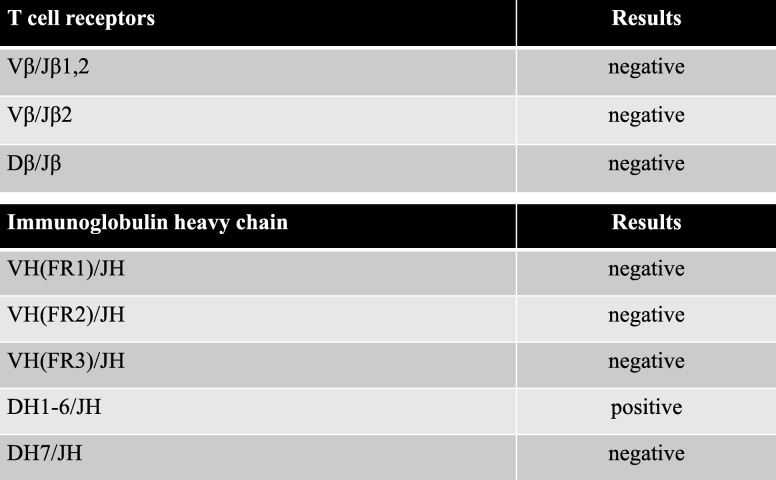
Recombination of immunoglobulin heavy chain and T cell receptors by polymerase chain reaction test

## Discussions

Mediastinal thyroid was present in 128 of 4142 (3.09%) patients with a mediastinal mass in Japan [3]. Furthermore, among patients with mediastinal thyroid tissue, ectopic thyroid tissue is extremely rare [4]. A defect in embryological development is believed to be a factor in the development of ectopic thyroid tissue. The genes associated with thyroid morphogenesis such as *NKX2-1*, *NKX2-5*, *PAX8*, *FOXE1*, and *thyroid-stimulating hormone receptor* (*TSHR*) have been reported to be involved in thyroid dysgenesis [9]. Studies comparing the gene expression of the normal thyroid with that of the ectopic thyroid gland found that ectopic thyroid glands showed differential gene expression [10].

The association of ectopic thyroid tissue with malignant transformation is uncommon, with the most common malignancy being papillary carcinoma [5]. Primary ectopic thyroid B cell lymphoma arising from an ectopic thyroid in the mediastinum is extremely rare, with only 1 reported case in 2009 [11]. The MALT lymphoma arising in association with ectopic thyroid in our patient, has, to our best knowledge, not been previously reported.

CT and MRI are useful for detecting incidental ectopic thyroid tissue. Ectopic thyroid tissue usually shows intense attenuation on contrast-enhanced CT [5]. On MRI, ectopic thyroid tissue has displayed increased signal intensity on both the T1- and T2-weighted images [5]. A mediastinal mass with DLBCL shows heterogeneous enhancement on CT. On MRI, a mediastinal mass with DLBCL shows hypointensity in fat on T1-weighted images, but isointensity in fat on T2-weighted images on MRI [12]. In our patient, CT showed early poor enhancement and delayed enhancement, especially in the central area. MRI showed low intensity on T1-weighted and high intensity on T2-weighted imaging. The histopathological examination showed abundant lymphocytes predominantly in the peripheral area. Although the detailed mechanism is unclear, ectopic thyroid tissue associated with MALT lymphoma might account for the imaging findings in our case.

Patients with ectopic mediastinal tissue are often asymptomatic and functionally euthyroid at presentation [13, 14], whereas, most patients with thyroid lymphoma are thought to show a rapidly enlarging goiter [15]. Dysphasia, dyspnea, hoarseness, Horner syndrome, and superior vena cava syndrome can occur if the mass enlarges to compress the surrounding tissues. In our patient, the lymphoma was manifested as an ectopic small solitary nodule without signs of compression, because the patient had a small MALT lymphoma, which is an indolent lymphoma.

DLBCL makes up about 50 to 80% of primary thyroid lymphomas, followed by extranodal marginal zone lymphomas, which include MALT and which account for about 30 to 40% [8, 15]. Pre-existing chronic thyroiditis is generally a risk factor for thyroid-associated MALT lymphoma [16]. The incidence of primary thyroid lymphoma in patients with Hashimoto thyroiditis has been reported to be 16 cases/year per 10,000 persons, which is much higher than the incidence of primary thyroid lymphoma in the Japanese general population, which is 0.02 cases/year per 10,000 persons [8, 17]. Our case had the features of chronic thyroiditis in ectopic thyroid tissue, which could lead to MALT lymphoma. Meanwhile, although FDG-PET imaging showed uptake in the cervical thyroid, ultrasound did not reveal pseudocysts or hypoechoic areas, which are features highly suggestive of lymphoma. Generally, differentiating chronic thyroiditis from thyroid lymphoma by FDG-PET is difficult [15]. In our case, a fine-needle aspiration biopsy of the cervical thyroid that was performed postoperatively revealed chronic thyroiditis, but not lymphoma. The postoperative level of sIL-2R was also normal. Therefore, additional resection of the cervical thyroid was not performed. However, we plan ongoing careful monitoring of the patient, and a large bore needle biopsy or excisional biopsy of the cervical thyroid might be performed if features highly suggestive of thyroid lymphoma develop.

Since the preoperative definitive diagnosis of a mediastinal mass is difficult to obtain, surgical resection is the general option. In our case, it was unclear preoperatively that the mass was ectopic thyroid tissue, in addition to malignant lymphoma associated with ectopic thyroid. The cervical approach is sufficient for the large majority of patients with goiters [4, 18], although extremely large goiters of 15–20 cm require a sternotomy [4]. In our patient, the size of the mass was 1.5 cm, and it was located anterior to the trachea in the superior mediastinum. Therefore, the mass was safely and completely resected via the cervical approach.

The effective treatment of thyroid lymphoma depends upon the tumor type and extent of the disease [15]. Localized MALT lymphoma can be effectively treated with local therapy alone [15]. Radiotherapy of the involved site is the favored choice for localized MALT lymphoma, as surgery alone cannot be considered if the resection margins are positive [19]. Our patient presented with a small solitary nodule, and first, we had to obtain the diagnosis. Therefore, we chose surgery and obtained a negative margin for the specimen, which was diagnosed as localized stage IE MALT lymphoma. Therefore, postoperative radiation therapy and systemic therapy were not undertaken. The 5-year overall survival rate of primary thyroid lymphoma has been reported to be 85%, and for patients with localized disease, the 5-year overall survival is 89% [8]. Thus far, our patient has not developed recurrence, and careful monitoring is ongoing.

## Conclusion

We reported a rare case of MALT lymphoma associated with chronic thyroiditis in ectopic mediastinal thyroid tissue. This case presents a new differential diagnosis associated with ectopic mediastinal thyroid tissue.

## Abbreviations

CD: Cluster of differentiation
CT: Computed tomography
FDG-PET: 18F-fluoro-deoxyglucose positron emission tomography
*FOXE1*: Forkhead box E1
MALT: Mucosa-associated lymphoid tissue
MRI: Magnetic resonance imaging
*NKX*: NK homeobox
*PAX8*: Paired box 8
sIL-2R: Serum interleukin-2 receptor
*TSHR*: Thyroid-stimulating hormone receptor
